# Medical management of victims contaminated with radionuclides after a “dirty bomb” attack

**DOI:** 10.1186/s40779-018-0174-5

**Published:** 2018-08-06

**Authors:** Alexis Rump, Benjamin Becker, Stefan Eder, Andreas Lamkowski, Michael Abend, Matthias Port

**Affiliations:** Bundeswehr Institute of Radiobiology, Munich, Germany

**Keywords:** Medical NRBC protection, Radiological emergency, Dirty bomb, Combined injuries, Radionuclide incorporation, Decorporation therapy

## Abstract

A wide spectrum of scenarios may lead to radiation incidents and the liberation of radioactive material. In the case of a terrorist attack by a “dirty bomb”, there is a risk of mechanical and thermal trauma, external irradiation, superficial contamination and incorporation of radioactive material. The first treatment priority must be given to the care of trauma patients with life-threatening injuries, as the health effects of radiation occur with latency. Radionuclide incorporation will lead to a longer-lasting irradiation from inside the body, associated with a higher risk of stochastic radiation effects (e.g., occurrence of tumors) in the long run. It must be expected that victims with potentially incorporated radionuclides will far outnumber trauma patients. The elimination of radionuclides can be enhanced by the administration of decorporation agents such as (Ca) Diethylenetriaminepentaacetic acid (DTPA) or Prussian blue, reducing the radiological burden of the body. There is still no consensus whether decorporation treatment should be started immediately based only on a suspicion of radionuclide incorporation (“urgent approach”) or if the results of internal dosimetry confirming the necessity of a treatment should be awaited, accepting the delay caused by the measurements and computations (“precautionary approach”). As the therapeutic effectiveness may be substantially decreased if treatment initiation is delayed only by several days, depending on the radionuclide, the physicochemical properties of the compounds involved and the route of absorption, we favor an “urgent approach” from a medical point of view. In doubt, it seems justified to treat victims by precaution, as the adverse effects of the medication seem minimal. However, in the case of a high number of victims, an “urgent treatment approach” may require a large number of daily doses of antidotes, and therefore, adequate investments in preparedness and antidote stockpiling are necessary.

## Background

A wide spectrum of scenarios may lead to radiation incidents and the liberation of radioactive material. The use of a nuclear weapon, an accident in a nuclear facility, as in Chernobyl, or the crash of a satellite with radioactive inventory can cause a large-scale disaster. Nevertheless, these scenarios are very different. Whereas a nuclear weapon attack would cause a large number of mechanically injured and burned victims in addition to irradiation [1], an incident in a nuclear facility would primarily lead to a large-scale contamination by fallout [2]. In Germany, an accident in a poorly maintained nuclear facility abroad is the most likely scenario. Currently, Belgian power plants are a topic of great public concern in the German population. In the Belgian border region, German authorities have even begun to distribute potassium iodide tablets to the population in case of a nuclear accident. The expected protracted dynamic of such an event would probably permit the civil protection authorities to respond to the situation, and besides the possibility of heavily injured victims inside or very close to the plant, a large number of cases of acute radiation sickness is highly unlikely. Accidents caused by the damage of installations where radioactive materials are stored must also be considered in military deployments and combat situations (Nuclear, biological and chemical - release other than attack, NBC-ROTA) [3]. Such dangerous installations may be damaged intentionally or unintentionally due to a lack of information about the stored materials.

Radiation accidents of different scales may happen as working accidents in nuclear power or recycling plants and in other industrial or research facilities [4]. Except several well-known events (e.g., the accident in Tokaimura) [5], most accidents involve a single or only a few workers and are fortunately of a small scale, for example, localized injuries contaminated with radioactive material. Moreover, in work-related accidents, the implied radionuclides are known, and the “radiological situation” can be relatively easily assessed from the beginning.

Lost radiation sources are also a topic of great concern. The removal of a radiotherapy device containing cesium-137 from an abandoned clinic in Goiania, Brazil, in 1987 and the distribution of the radioactive material among friends and neighbors is probably the most prominent case. Twenty people were admitted to the hospital, 4 of whom died within 4 weeks [6]. Even today, it happens that devices containing radioactive materials (e.g., americium-241) that have not been disposed of correctly are found in scrapyards by chance, but fortunately, inadvertent injuries are uncommon.

Nuclear or radiological terrorism is another hazard. The construction of an improvised nuclear device (IND) certainly requires substantial financial means and particular technical capabilities. Fissile material must at first be obtained and possibly chemically treated before it can be used for weapon purposes. In addition, even the construction of a device based on the simpler “gun-shot design”, compared to the more demanding “implosion design” [7], is not within the reach of every terrorist group. Nevertheless, the danger of an IND must not be totally discarded, as shown by the 1995 sarin attack in Tokyo by the Japanese Aum Shinrikyo doomsday cult who had substantial financial funds and employed highly skilled scientists [7, 8].

Compared to improvised nuclear devices, the construction of a radiological dispersal device (“dirty bomb”) combining conventional explosives and radioactive material is certainly much easier. It is not possible to predict the concrete means and the way a terrorist attack would be done, but radionuclides widely used in industry, medicine or research are of particular concern (Table 1) [9, 10].

**Table 1:** Radionuclides of concern that might be used in the construction of a “dirty bomb”. [9] Source for physical and effective half-life values [10]

Item	Am-241	Cf-252	Co-60	Cs-137	I-131	Ir-192	Pu-238	Pu-239	Ra-226	Sr-90	U-235
Radiation emitted	α, γ	α, n	β, γ	β, γ	β, γ	β, γ	α	α	α, γ	β	α, γ
Physical half-life	432 a	2.65 a	5.3 a	30 a	8 d	73.8 d	87.7 a	24 10^3^ a	1600 a	28.2 a	7 × 10^8^ a
Effective half-life	45 a	2.5 a	1.6 a	109 d	7.5 d	–	50 a	50 a	44 a	4.6 a	15 d

“-” Not available; a: years; d: days

Scenario techniques permit us to develop alternative plausible events used for capacity-based planning, but it is not possible to assign probability values to individual defined incidents [11]. The targets of a “dirty bomb” attack might greatly differ depending on the effects sought. One possible objective might be to cause indirect economic damage as a consequence of long-lasting radioactive contamination of critical infrastructure. A risk and economic analysis of “dirty bomb” attacks on the ports of Los Angeles and Long Beach has shown that the shutdown of port operations could result in a total loss of tens of billions of dollars [12]. Nevertheless, the psychological impact may be greater in the case of a detonation in a densely populated city area. The number of victims would certainly depend on the means used as well as the location and time of the attack. The federal interagency community in the US has developed fifteen all-hazard scenarios, among them a radiological attack [13]. This scenario describes the almost simultaneous detonation of “dirty bombs” contaminated with cesium-137 at 3 locations and causing 180 fatalities, 270 injured and 20,000 radioactively contaminated people at each site. We examined the possible targets of attack in Germany (e.g., Christmas markets, fun fairs, famous shopping streets) and came to the conclusion that for a “dirty bomb” attack, planning for 60,000 people potentially contaminated with radioactive material seems quite reasonable [14].

In the case of a “dirty bomb” attack, some patients will be expected to suffer blast injuries with mechanical trauma due to fragments and burns. External irradiation by ionizing radiation may also occur. Nevertheless, most hypothetical scenarios described are not associated with a risk of acute radiation sickness development, in contrast to early fallout exposure after a nuclear detonation [15, 16]. However, external radioactive contamination would probably occur on an extended surface and may affect a large number of people, many more than the victims of mechanical or thermal trauma. External contamination may always lead to the incorporation of the radionuclide(s). Except special cases such as the Litvinenko poisoning [17], the incorporation of radionuclides is usually not expected to induce an acute radiation sickness [18] but may cause stochastic health effects (e.g., cancer) in the long run. Moreover, external contamination may endanger rescue personnel by secondary contamination.

### Medical emergency measures and treatment priorities

**Fig. 1:**
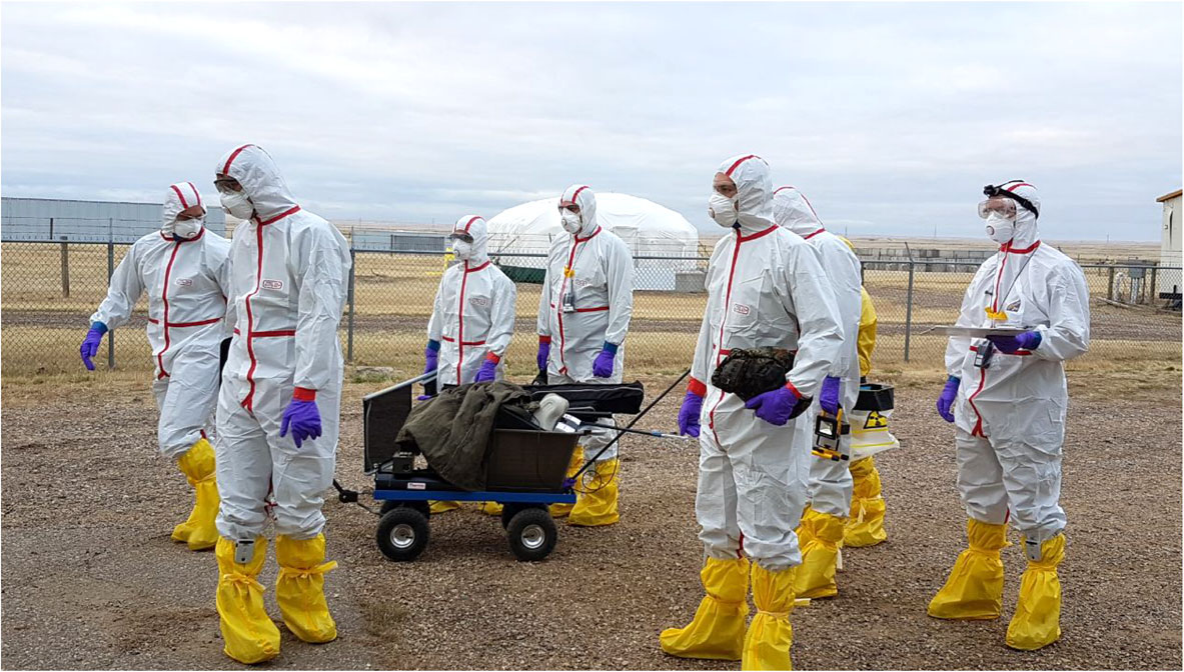
The protective equipment of the medical task force for nuclear and radiological emergencies of the Bundeswehr Institute of Radiobiology. Light equipment is sufficient to protect against radioactive contamination but permits good patient care

Hazmat (hazardous materials) incidents require a particularly prudent approach, but unjustified fears must not lead to avoidable delays in the rescue and treatment of patients. As ionizing radiation is not visible, the local dose rate should be assessed using radiation measuring/sensing devices. In the case of elevated radiation dose levels, the victims must be rapidly evacuated from this area. Victims only externally irradiated do not represent any particular danger to rescuers or hospital staff. The same hygiene standards apply as for all patients [19]. However, victims externally contaminated with radioactive material (e.g., contaminated dust from the detonation) may contaminate and endanger their environment. Members of rescue teams must protect themselves from secondary contamination and possible radionuclide incorporation. This includes wearing protective clothing, respiratory protection (particle filter mask protecting also the eyes) and gloves. A heavy, full NRBC (Nuclear, Radiological, Biological and Chemical) protective equipment is certainly adequate protection but is not absolutely required for the care of patients contaminated with radionuclides, in contrast to patients who have contacted highly toxic chemicals (e.g., nerve agents or corrosive substances) [19, 20]. In particular, it is sufficient to wear simple surgical gloves (preferably 2 pairs) to avoid unnecessary hindrances when handling patients and performing medical procedures (Fig. 1).

Past experiences from accidents with radioactivity indicate that the dose rate emanating from contaminated victims is low and that emergency personnel are usually not endangered by external irradiation [19, 21]. Thus, fear of approaching contaminated patients, if wearing sufficient protective equipment, is not justified. Nevertheless, scenarios with patients hurt by shrapnel made of highly radioactive material are conceivable, and as a precaution, dose rate measurement is advisable.

In the case of combination injuries (mechanical/thermal injury + irradiation and/or radioactive contamination), it should be noted that mechanical trauma can cause an immediately life-threatening situation (e.g., tension pneumothorax, massive intra-abdominal bleeding), whereas acute radiation sickness develops with a latency ranging from days to weeks. Even prodromal symptoms such as nausea or vomiting may be seen only after several hours. Radionuclide incorporation and long-lasting internal irradiation will probably cause health effects only in the long run. As in every medical emergency, the principle “Treat first what kills first” applies. The preservation of the vital functions always has first priority. Thus, the initial triage must be done using the general rules of trauma care. The algorithms developed in the Prehospital Trauma Life Support (PHTLS) concept or the corresponding Advanced Trauma Life Support (ATLS) concept for emergency rooms give a good guidance for assessing and treating trauma patients [22]. These concepts are based on sound principles and use a uniform and simple terminology suited to facilitate communication. The PHTLS concept also forms the medical basis of Tactical Combat Casualty Care (TCCC). Meanwhile, PHTLS course formats are increasingly used to train military emergency physicians in several countries, including Germany [23]. Applying triage systems specific to radiation accidents does not make sense at this very early stage but should be reserved to retriage the patients at a later time [24].

Victims without serious injuries and no indication for urgent medical measures should be fully decontaminated first by undressing and rinsing the body. Decontamination must be ascertained by measurement before starting further medical checkup or treatment. Decision making is more complex in the case of seriously mechanically injured patients, as the urgency of a surgical treatment must be weighed against a carryover of radioactive contamination into rescue vehicles, emergency rooms and operation theatres. The issue has been encapsulated in the statement of a senior emergency physician of the city of Vienna with a radiological background: “The optimal balance between medical diagnostic and therapeutic measures and the requirements of radiation protection is always the most desirable” [25]. Different strategies may be applied, depending on the preparedness of the medical facilities to admit radio-contaminated patients without prior full decontamination as well as the total number of patients versus treatment capacities. In the case of a patient with an urgent vital indication for surgery (e.g., massive intra-abdominal hemorrhage), at least a provisional decontamination should be performed by undressing the victim before transportation. This simple and rapid procedure can be expected to remove a large part of the radioactive contamination that has deposited on the clothes (70–80%). If possible, plastic sheets should be placed under and on the stretcher to facilitate the decontamination of the equipment afterwards. Patients should also be covered to minimize secondary contamination of the environment. The receiving treatment facility must be informed about the radioactive contamination, so that admission can be prepared. Some hospitals are organized to admit radioactively contaminated patients and have developed protocols to rapidly prepare emergency rooms or operation rooms for that purpose.

Although radiological doses below the threshold levels for an acute radiation sickness will not cause clinical effects in the short run, stochastic effects and health impairment in the long run must be expected. All victims should therefore be examined by a physician with specific knowledge on radiation accidents and get advice. The dose absorbed can be estimated by repeated differential blood counts or with more precision by blood exams, referred to as biodosimetry [26]. The gold standard consists in quantitating dicentric chromosomes in lymphocytes caused by misrepair of ionizing radiation-induced DNA double-strand breaks (chromosomes normally have only one centromere). Dicentric chromosomes are highly specific for the exposure to ionizing radiation. Further procedures use the quantitation of micronuclei or translocations or are based on gene expression. The dose that will be absorbed by internal irradiation due to the incorporation of radionuclides can be quantified by whole-body counting or excretion analysis, depending on the nuclide and the kind of radiation emitted, followed by internal dosimetry calculations.

**Fig. 2:**
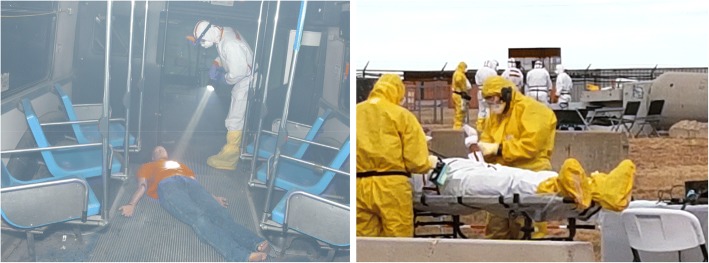
The medical task force for nuclear and radiological emergencies of the Bundeswehr Institute of Radiobiology in action during the exercise Precise Care 2017

The Bundeswehr Institute of Radiobiology has a task force for nuclear and radiological emergencies that may be deployed in Germany or abroad to assist with technical equipment. It has a limited stock of specific antidotes and is staffed with specialized physicians to give medical advice on managing patients at the site of the incident (Fig. 2). Moreover, the institute offers a wide spectrum of diagnostic capabilities and is integrated with international networks of institutions specialized in radiological emergencies, permitting it to fulfill coordination activities [27].

### Internal dosimetry

When radionuclides are incorporated, they distribute in the body and are deposited in different tissues and organs, depending on their chemical nature. Their elimination from the body is variable and is done by the same excretion mechanisms as for stable (nonradioactive) chemical entities, as well as by radioactive decay The effective half-life, depending on both processes, ranges from days (iodine-131: 7–8 days) to months (cesium-137: 109 days) to decades (plutonium-239: 50 years) [10]. The radioactive decay of the radionuclides leads to a protracted and, in part, long-lasting irradiation of the body from the inside. Except in rare cases [17], past experience indicates that the doses absorbed within a short time frame are much below the threshold of an acute radiation sickness [18]. However, the continuing irradiation enhances the risk of stochastic health effects such as the future occurrence of tumors and other pathologies [28, 29]. The radiation exposure is usually quantitated by the committed effective dose, which is defined as the total effective dose due to radionuclide incorporation absorbed over 50 years after the incident (70 years for children). This dose cannot be directly measured by a sensing device, as the dose rate of radiation emanating from a source in the environment can. The determination of the committed effective dose requires the measurement of radioactivity in the body by whole-body counting or radionuclide excretion measurements in urine or feces, followed by internal dosimetry calculations. Computations are based on complex physiologically oriented kinetic models describing the absorption, distribution and elimination of a defined radionuclide, as used in toxicology [30], in combination with a dosimetric model describing the absorption of energy in the different organs and tissues due to the radioactive decay [31]. Taking into account the kind of radiation (alpha, beta or gamma) and the relative sensitivities of the different tissues to radiation, the integration over the considered time interval (usually 50 years) gives the committed effective dose (unit mSv, millisievert) as a metric of the stochastic health effects.

The International Commission on Radiological Protection (ICRP) and the National Council on Radiation Protection (NCRP) have developed biokinetic models to describe absorption processes: the respiratory tract model [32], the gastrointestinal tract model [33] and the wound model [34, 35]. These models are generic and may be applied to all radionuclides. On the other hand, the systemic models describing disposition after absorption and elimination processes are specific for a radionuclide or a group of nuclides. The different modules can be combined to result in a complete model suitable for a particular case (the most suitable combination for a mechanically injured patient after a “dirty bomb” attack would probably be the respiratory tract model + the wound model + the systemic model for the identified radionuclide). For all models, the ICRP proposes parameters based on the best evidence available. As the mathematics of the models is very complex due to the number of compartments, software packages are available for internal dosimetry computations. At the Bundeswehr Institute of Radiobiology, we use Integrated Modules for Bioassay Analysis (IMBA) software for that purpose.

**Fig. 3:**
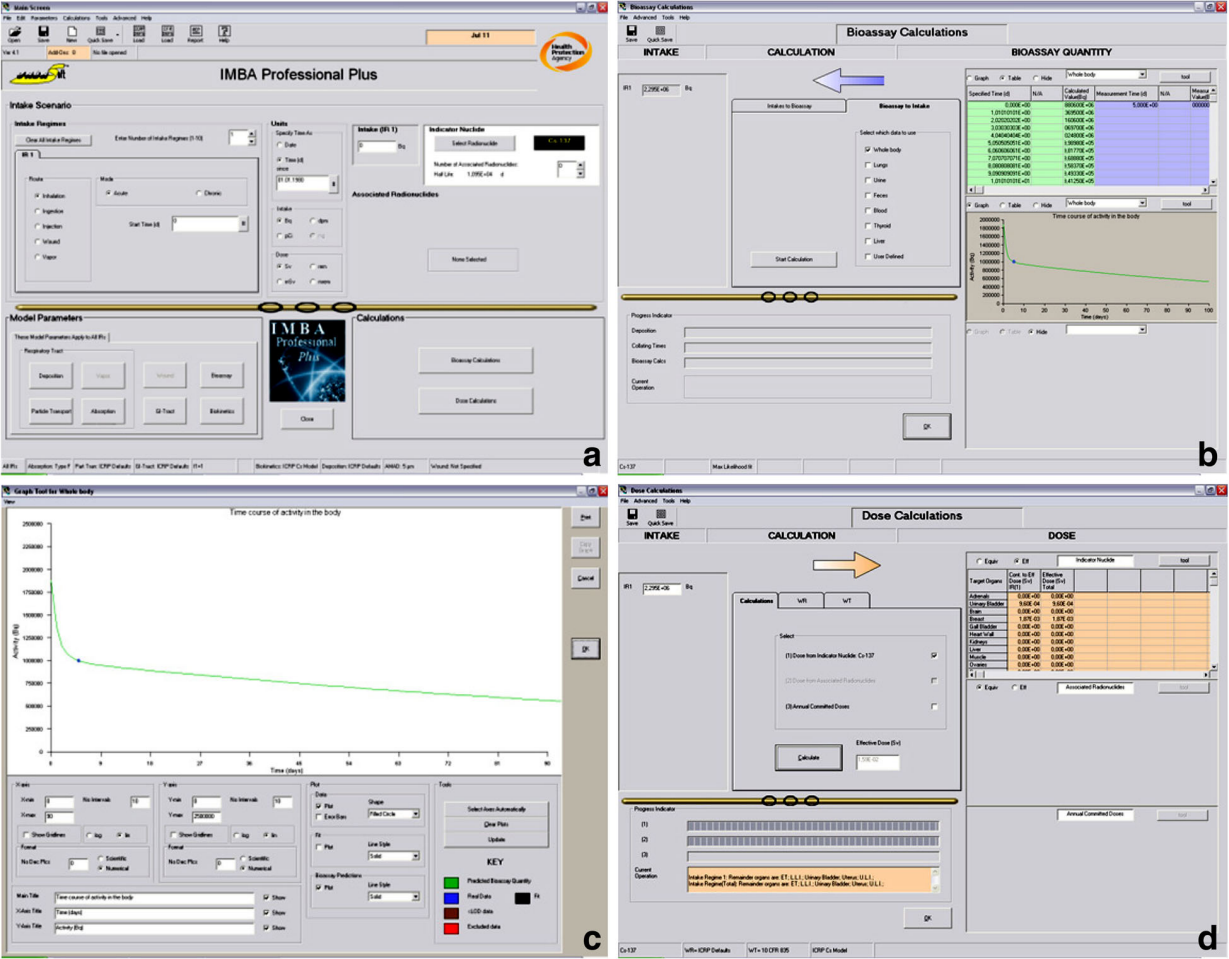
The different steps of internal dosimetry computations using IMBA software. **a** Radionuclide selection; **b** Computation of the initial activity intake from the measured activity; **c** Representation of the time course of activity in the body; **d** Computation of the committed effective dose from the initial activity intake. IMBA. Integrated Modules for Bioassay Analysis

Internal dosimetry requires first the measurement of radioactivity in the body at the time of the examination. In the second step, the initial radioactivity intake at the time of the incident is calculated using the most suitable biokinetic model. In the last step, this activity is used to compute the committed effective dose (Fig. 3). Depending on the method (whole-body counting, radio-toxicological method), the availability of the measurement equipment, the number of patients and logistics, internal dosimetry may take days or even weeks before results are available.

### Mechanisms and principles of decorporation therapy

Drug stockpiles for nuclear and radiological emergencies usually include Granulocyte-colony stimulating factor (G-CSF), potassium iodide and the decorporation agents diethylenetriaminepentaacetic acid ((Ca)DTPA and (Zn)DTPA), as well as Prussian blue (ferric hexacyanoferrate) [36, 37]. In the blood or extracellular space, (Ca)DTPA exchanges the less firmly bound calcium ion for many metal radionuclides, among them plutonium-239 and americium-241, and speeds up their renal excretion (Table 2) [10, 37, 38]. It must be mentioned that the assessment of therapeutic efficacy is not conclusive for all radionuclides, and recommendations vary. (Ca)DTPA is usually injected intravenously but may also be administered by inhalation. Its oral bioavailability is poor [37]. Prussian blue, which is administered orally, binds cesium-137, which is secreted through the bile into the gut, and thus prevents its reabsorption into the blood and enhances its elimination through the feces [39, 40]. In some countries, chelators with sulfhydryl-groups, such as DMPS (2, 3-Dimercapto-1-propanesulfonic acid) or DMSA (dimercaptosuccinic acid), are also considered necessary for radiological emergencies [41]. These may, for example, be used to treat polonium-210 contamination, although their effectiveness is doubtful, and animal results do not give a conclusive picture on the best decorporation agent for this indication [17, 42].

**Table 2:** The two decorporation agents that are essential for nuclear/radiological emergencies and the radionuclides whose excretion can be enhanced

Antidote	Radionuclides
(Ca)DTPA	Americium, Californium, Cerium, Chrome, Cobalt, Curium, Erbium, Europium, Iron, Gallium, Iridium, Lanthanum, Manganese, Plutonium, Praseodymium, Promethium, Ruthenium, Samarium, Scandium, Thorium, Ytterbium, Yttrium, Zinc, Zirconium; Mixture of fission products
Prussian blue	Cesium, Thallium, Indium; Mixture of fission products

Once the radionuclide has been identified, the selection of the right antidote usually is not an issue (Table 2). However, there is no consensus when to start the treatment. According to the “precautionary approach”, internal dosimetry results should be awaited and decorporation treatment only started if a relevant committed effective dose has been confirmed (> 20 mSv or > 200 mSv, depending on the standard that has been set) [38, 43]. This approach emphasizes that it is not justified to expose a patient to the potential side effects of a medication as long as the actual need is still unclear. According to the “urgent approach”, it is prudent and advisable to start decorporation treatment immediately, even if radionuclide incorporation is only suspected [10, 38]. Treatment should be discontinued once internal dosimetry results are available and the committed effective dose has been shown to be not relevant. Simulations based on the biokinetic models help to quantitatively assess the real impact of the initiation time of the treatment on its effectiveness.

**Fig. 4:**
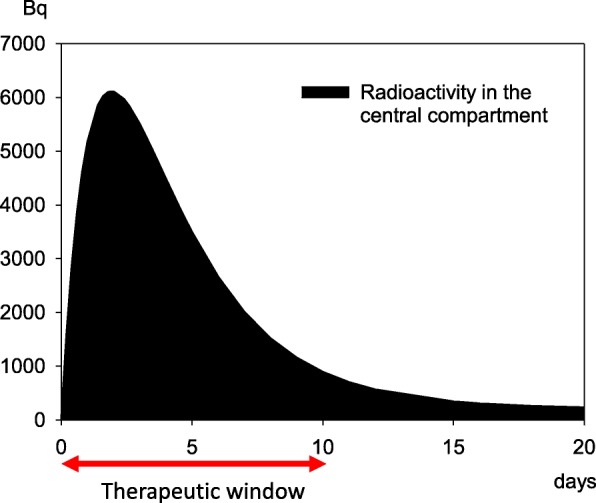
Time course of radioactivity in the central compartment [44] (blood, extracellular space) emanating from a wound contaminated with 37 kBq (1 μCi) of plutonium-239 as a soluble compound. Activity falls to low values after approximately 10 days. The decorporation agent (Ca) DTPA is distributed mainly in the extracellular space, where it can bind plutonium. Thus, treatment must start within 10 days to be highly effective (“therapeutic window”)

Simulating the time course of radioactivity in the central compartment (blood and extracellular space) resulting from a wound contaminated with a highly soluble plutonium-239 compound shows that activity rapidly increases till the end of the second day and decreases thereafter, reaching very low values after approximately 10 days (Fig. 4) [44]. During this time, plutonium-239 is redistributed in “deep” compartments such as bone and liver. (Ca)DTPA, the agent of choice for decorporation of plutonium-239, is distributed mainly in the extracellular space, where it can bind plutonium [38, 43]. Thus, there is a window of opportunity during the first 10 days after the incident to achieve a high therapeutic effectiveness, and even during this time, a delay of treatment initiation will be associated with a decrease in effectiveness as the total amount of plutonium that can be rapidly eliminated decreases. Once plutonium has entered the “deep” compartments, it cannot be bound by (Ca) DTPA in substantial amounts. These pharmacokinetic considerations are supported by the computations of the committed effective doses: Assuming that 37 kBq (1 μCi) of plutonium-239 is contaminating the wound, leading to a radiological dose of 823 mSv without treatment, the dose can be reduced to 10 mSv if treatment is started after 2 h, 57 mSv if it is started after 1 day, but only 501 mSv if treatment initiation is delayed up to the 10th day after wounding [44].

**Fig. 5:**
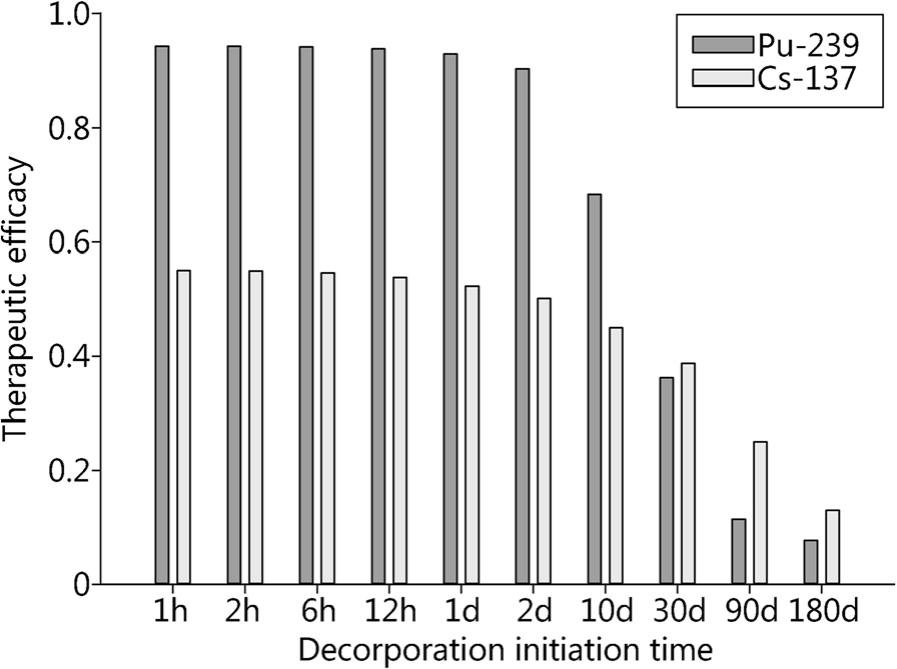
Efficacy of a decorporation treatment using (Ca)DTPA or Prussian blue after the acute inhalation of a poorly soluble compound containing plutonium-239 [45] (e.g., plutonium oxide) or a soluble compound containing cesium-137 (e.g., cesium chloride), depending on the time after the incident the treatment is initiated. Treatment duration is assumed to be 90 days. Efficacy = 1 – (dose with treatment / dose without treatment)

In the case of plutonium-239, (Ca)DTPA treatment is highly effective if started early (Fig. 5) [45]. If treatment is delayed, the decrease in therapeutic effectiveness is particularly rapid if the invasion kinetic of plutonium is fast. If treating americium-241 incorporation using (Ca)DTPA, the maximum therapeutic effectiveness is lower than for plutonium-239, even if started very early, but the decrease in effectiveness if treatment is delayed occurs at a slower rate. The same applies to the decorporation of inhaled cesium-137 by Prussian blue treatment (Fig. 5). Therapeutic effectiveness may be increased by extending the duration of treatment. However, the possibilities to compensate for a delay in treatment initiation by extending treatment duration are limited [45].

The maximum therapeutic effectiveness and the consequences of a delay in treatment initiation depend on the radionuclide, the invasion pathway and the physicochemical properties, in particular the solubility of the compounds involved [45]. So a clear-cut time slot for the initiation of decorporation treatment cannot be given in general terms. Depending on the situation, it seems to be in the range of hours to several days.

To assess the benefit/risk ratio of a treatment, the importance and incidence of the adverse effects of the medication must be considered. For (Ca) DTPA, side effects such as gastrointestinal distress, chills, fever, pruritus and muscle cramps have been reported. In the long run, a depletion of zinc and, to a lesser extent, manganese may occur. However, after over 4500 administrations of (Ca) DTPA and 1000 administrations of (Zn) DTPA at the recommended dosages, serious clinical complications have not been observed [46]. A survey of cases treated with (Ca)DTPA from 1970 to 2003 in France at facilities of the Commissariat à l’Énergie Atomique (CEA) and Compagnie Générale des Matières Nucléaires (COGEMA) (total of 1158 injections to 469 patients) revealed a good tolerability of the treatment [47].

Prussian blue may bind electrolytes in the gut and cause hypokalemia. Only slight electrolyte disturbances without clinical consequences were observed among the patients treated with Prussian blue in Goiânia [40]. Light to moderate constipation was reported in 10 of 46 patients [40]. These patients responded well to diet control and laxatives [6]. Due to its chemical structure, the liberation of cyanide ions from Prussian blue has been a concern. It was, however, shown that the maximal amount of cyanide that is liberated under physiological conditions is very small compared to toxic doses [48].

As it seems that there are few adverse side effects of (Ca)DTPA and Prussian blue in short-term treatments [38, 42, 47], the “urgent approach” strategy seems prudent and has been adopted at the Bundeswehr Institute of Radiobiology. However, depending on the scale of the incident, this strategy may require a very large number of daily doses of antidotes and requires corresponding investments in the preparedness level by the responsible authorities [14].

### The health value of decorporation therapy

A further difficult issue relates to the true utility of a decorporation treatment expressed not as a reduction of the radiological dose absorbed but as lifetime saved. As already mentioned, the incorporation of radionuclides may particularly causes stochastic health effects (e.g., cancer) in the long run. Averaged over all age groups, an additional dose of 1 mSv is associated with a statistical loss of 0.42 day over a lifetime [49, 50]. We have previously shown that using an “urgent approach” could save more total lifetime than a treatment based on a “precautionary approach” [14].

Nevertheless, a statistical lifetime extension of 0.42 day per mSv appears quite a short time, particularly if considering the values often used to decide on a treatment indication (20 mSv corresponds to 8.4 days saved, and 200 mSv to 84 days). Although this is a very controversial and even philosophical question, it might be justified to ask how meaningful it would be to extend the life of an elderly person having reached his/her full “natural life span” (concept of the philosopher Callahan) [51, 52] by only several days. However, in our case, the issue should be viewed differently: “Premature deaths”, i.e., death before living a “natural life span” [51], must be expected in some individuals, whereas other individuals will remain healthy, as the figure of 0.42 day/mSv is a mean statistical value and does not uniformly apply to all individuals having absorbed a radiological dose. The prevention of these “premature deaths” may be viewed as one justification for decorporation treatment.

It is also interesting to assess the utility issue from the perspective of health economics and to compare the benefits with the costs. As the utility of a decorporation treatment is expressed as statistical lifetime saved, the concept of “value of a statistical life” can be used to quantitate the benefits in a monetary unit [53]. This is a method sometimes used when making decisions on investments in environmental technologies or transportation infrastructure projects to improve safety [54]. We applied this concept to a scenario involving the incorporation of cesium-137 and showed that a decorporation treatment with Prussian blue was not only effective but also efficient from a microeconomic point of view at the level of a single patient [50]. It must be emphasized that this analysis is based on values specific to Germany (e.g., costs of medication), and these results cannot be automatically applied to other countries. The issue of how to precisely define the cutoff point to decide on the indication of decorporation treatment remains.

## Conclusions

Nuclear and radiological scenarios may greatly differ in nature and scale. In the case of a “dirty bomb” attack, patients with mechanical and thermal trauma must be expected, in addition to a much larger number of victims having potentially incorporated radionuclides. Therefore, besides a proper technical and medical management at the site of the incident, a clear strategy for dealing with a large number of patients who may be internally contaminated is mandatory. Early initiation of treatment, even based only on a suspicion of radionuclide incorporation, according to the “urgent approach” strategy seems to be sound from a medical point of view but requires a demanding antidote-stockpiling policy.

## Abbreviations

ATLS: Advanced trauma life support
CEA: Commissariat à l’Énergie atomique
COGEMA: Compagnie Générale des Matières Nucléaires
DMPS: 2,3-dimercapto-1-propanesulfonic acid
DMSA: Dimercaptosuccinic acid
DTPA: Diethylenetriaminepentaacetic acid
G-CSF: Granulocyte-colony stimulating factor
Hazmat: Hazardous materials
ICRP: International Commission on Radiological Protection
IMBA: Integrated modules for bioassay analysis
IND: Improvised nuclear device
mSv: Millisievert
NBC-ROTA: Nuclear, biological, chemical-release other than attack
NCRP: National Council on Radiation Protection
NRBC: Nuclear, radiological, biological and chemical
PHTLS: Prehospital trauma life support
TCCC: Tactical combat casualty care
